# Final-year medical students’ competence profiles according to the modified requirement tracking questionnaire

**DOI:** 10.1186/s12909-021-02728-2

**Published:** 2021-06-05

**Authors:** Elena Zelesniack, Viktor Oubaid, Sigrid Harendza

**Affiliations:** 1 III. Department of Internal Medicine, University Medical Center Hamburg-Eppendorf, Martinistr. 52, D-20246 Hamburg, Germany; 2 German Aerospace Center (DLR), Hamburg, Germany

**Keywords:** Competence, Final year, Medical specialty, Residency, Postgraduate medical education, Undergraduate medical education

## Abstract

**Background:**

Undergraduate medical education is supposed to equip medical students with basic competences to select any specialty of their choice for postgraduate training. Medical specialties are characterized by a great diversity of their daily work routines and require different sets of competence facets. This study examines the self-assessed competence profiles of final-year undergraduate medical students and their specialty choice for postgraduate training. Students’ profiles, who wish to choose anaesthesiology, internal medicine, or paediatrics, are compared with the physicians’ competence profiles from these three disciplines.

**Methods:**

In this study, 148 volunteer final-year undergraduate medical students completed the modified requirement-tracking (R-Track) questionnaire for self-assessment of their competence profiles. The R-Track questionnaire contains 63 competence facets assigned to six areas of competence: “Mental abilities”, “Sensory abilities”, “Psychomotor & multitasking abilities”, “Social interactive competences”, “Motivation”, and “Personality traits”. The expression of the different competence facets had to be assessed on a 5-point Likert scale (1: “very low” to 5: “very high”). Additionally, socio-demographic data and the participants’ first choice of a medical speciality for postgraduate education were collected. We used analysis of variance (ANOVA) for mean score comparison of subgroups and least significant difference (LSD) tests for post hoc analysis.

**Results:**

The competence area with the highest rating was “Motivation” (3.70 ± 0.47) while “Psychomotor & multitasking abilities” received the lowest rating (3.34 ± 0.68). Individual facets of competence ranked from “In need of harmony” (4.36 ± 0.72), followed by “Tactfulness” (4.26 ± 0.64), and “Cooperation/Agreeableness” (4.24 ± 0.53) to “Risk orientation” (2.90 ± 0.92), “Mathematical reasoning” (2.87 ± 1.25), and “Sanctioning” (2.26 ± 0.93). The students’ competence profiles showed 100 % congruence with physicians’ competence profiles of the postgraduate specialty of their choice for internal medicine, 33.3 % for paediatrics, and 0 % for anaesthesiology.

**Conclusions:**

Undergraduate medical students could define their competence profiles with the modified R-Track questionnaire and compare them with the profile of their desired specialty for postgraduate training to discover possible learning gaps or to detect good specialty matches. A combination of students’ competence self-assessment with an external assessment of students’ facets of competence could provide curricular planners with useful information how to design learning opportunities for specific facets of competence.

**Supplementary Information:**

The online version contains supplementary material available at 10.1186/s12909-021-02728-2.

## Background

The goal of undergraduate medical education is to prepare medical graduates to start their postgraduate training in any medical specialty they wish to choose [1, 2]. To achieve this goal, many countries developed catalogues with basic learning objectives for undergraduate medical education [3–5]. The final year of undergraduate training or internship [6, 7], depending on the educational system [8], is supposed to facilitate the transition to residency and should prepare the students to work in clinical settings and get accustomed to the roles and responsibilities of a resident [9–11]. During the clinical rotations within the final year, the students increasingly take responsibility, gain experience, deepen their understanding of the areas of interest, and explore career options [6, 10, 12]. On the one hand, students have finished their basic education and can acquire relevant insights into their desired specialty for postgraduate training [6]. On the other hand, this experience can lead to the insight that personal interest and abilities are unsuitable for the previously desired specialty [11, 12].

The medical specialties are characterized by a great diversity regarding their everyday work routines, which require different competences. Interestingly, different personality profiles of physicians working in different specialties have been identified [13–15]. Furthermore, differences in non-technical attributes have been found between staff, trainees, and residency applicants within four surgical specialties [16]. In order to define medical specialty specific profiles, the Requirement-Tracking questionnaire (R-Track) was developed [17]. With R-Track, competence profiles for anaesthesiologists have been defined [18]. Within the internal medicine subspecialty of nephrology, we found different competence profiles for hospital-based nephrologist and nephrologists working in private practice [19]. In a pilot study, we defined competence profiles for 17 different medical specialties [20].

The individual choice of a medical specialty for postgraduate education is an important, often lifelong career decision [21, 22]. Most medical graduates make their final specialty choice early in their postgraduate years, only very few residents change their specialty during the first four years of training [23–25]. Choosing a specialty does often not include matching an individual’s competences with the competences required for a certain medical specialty. It rather focuses on several other factors such as academic performance in general, clinical experiences and clerkships, or applicants’ perceived benefits of a specialty with respect to a specialty’s potential income, prestige, work-life balance, or amount of patient contact [26–30]. For example, the National Resident Matching Program (NRMP) provides a specific algorithm that matches candidates for postgraduate education after interviews with the providers of residency programs according to a submitted Rank Order Lists (ROLs) which includes their preferences [31]. In general, medical graduates are not given the opportunity to identify their individual competence profile and match it with competence profiles required for certain medical specialties.

The R-Track could be useful for medical students near graduation to identify their own competence profiles close to finishing their undergraduate education and match it with competence profiles of medical specialties. Therefore, this pilot study addressed two research questions: (1) What do final-year medical students’ self-assessed competence profiles look like? (2) How do final-year medical students’ self-assessed competences profiles compare to the competence profiles of physicians from the students’ intended choice of specialty for postgraduate training?

## Methods

### Study design, participants and instrument

This survey of self-assessed competence profiles was part of a competence-based assessment of advanced undergraduate medical students in the physician’s role during a simulated first workday [32, 33]. Participation was voluntary and registration for one of the available 150 slots for participation occurred on a first come, first served basis. All final-year medical students, approximately 280, of the Medical Faculty of the University of Hamburg, Germany, were invited by email to participate in the assessment between October and December 2019. To depict medical competence profiles, 148 (52.8 %) volunteer final-year medical students of a 6-year undergraduate curriculum completed the R-Track (Requirement-Tracking) questionnaire before the simulation began. The R-Track questionnaire was conceptualized by Dr. Viktor Oubaid, psychologist at the German Aerospace Center. It was inspired by questionnaires like the Fleishman Job Analysis Survey (F-JAS) [34], and was originally designed to detect competence profiles of aviation and space personnel (e.g. airline pilots) as well as different medical specialties or professional groups [17]. The F-JAS as a job analysis instrument is usually used for the direct collection of skills and abilities (summarized as ‘competences’) that are relevant for the accomplishment of certain occupational tasks or activities [35]. It describes individual activities or compares entire fields of activity and can be used for all professions [35], while the R-Track was designed specifically for “safety industries” such as pilots, astronauts, physicians, and other health professionals, where personality traits and social interactive competences are of substantial relevance.

The R-Track questionnaire includes 63 questions, which are assigned to 6 areas of competence: “Mental abilities” (14 questions), “Sensory abilities (9 questions), “Psychomotor & multitasking abilities” (2 questions), “Personality traits” (12 questions), “Motivation” (5 questions), and “Social interactive competences” (21 questions). Competences represent the individually developed repertoire of abilities, skills, personality traits, and motivational aspects critical to effective and successful performance [36]. Questionnaires for job requirements should be able to differentiate between different occupations. Previous studies with the R-Track questionnaire revealed differences between medical specialties [17, 20], which were in line with the expected differences. For this study, we adapted the R-Track questionnaire to be used for self-assessment of the respective competence facets (Supplement 1). For this purpose, the questions for the individual items were rephrased to the first person singular. The final-year medical students were asked to assess the expression of the different competences on a 5-point Likert scale (1: “very low” to 5: “very high”). They were also asked to indicate their choice of a medical specialty for postgraduate education. Additionally, socio-demographic data were collected including age and sex. The Cronbach’s alphas of the scales varied on a medium level between .51 (“Psychomotor & multitasking abilities”), .55 (“Motivation”), .56 (“Personality traits”), .71 (“Sensory abilities”), .75 (“Social interactive competences”), and .82 (“Mental abilities”). This study was performed in accordance with the Declaration of Helsinki and the Ethics Committee of the Chamber of Physicians, Hamburg, confirmed the innocuousness of the study with consented, anonymized, and voluntary participation (PV3649). All participants provided informed written consent for participation in this study.

### Data processing

The R-Track questionnaire is presented in an online version. Participants in this study used an ID code in order to avoid the storage of personal data (name, age etc.). The resulting data where analysed in SPSS 25, using ANOVA procedures for mean score comparison of subgroups. Post hoc tests were carried out by using LSD tests. Differences were considered significant for p-values < 0.05. For comparison of participating students’ profiles with physician’s profiles from the medical specialties students wished to choose for postgraduate training, results from a previous R-track study with practicing physicians were used [20].

## Results

**Fig. 1:**
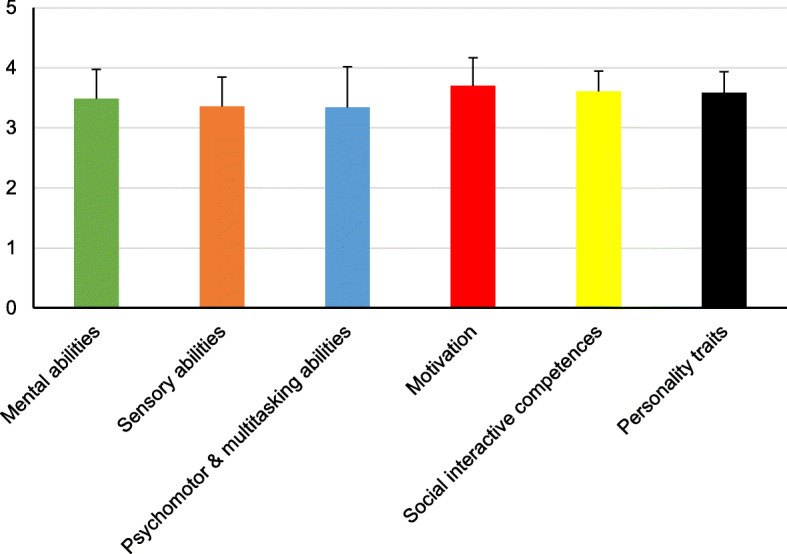
Self-assessed areas of competence

**Fig. 2:**
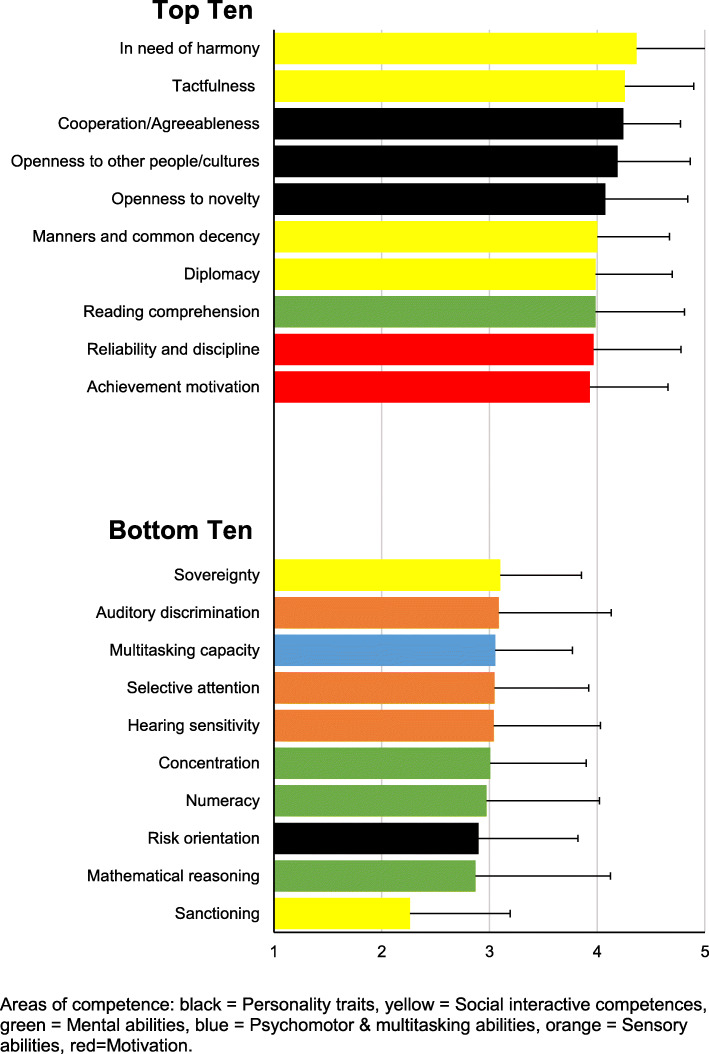
Top ten and bottom ten characteristics from the different areas of competence

All data sets of the 148 participants (64.9 % female) were fully completed and included in the analysis. Of the self-assessed areas of competence, participants reached the highest mean score for “Motivation” (3.70 ± 0.47), followed by “Social interactive competences” (3.61 ± 0.33), “Personality traits” (3.58 ± 0.35), “Mental abilities” (3.48 ± 0.49), “Sensory abilities” (3.36 ± 0.49), and “Psychomotor & multitasking abilities” (3.34 ± 0.68) (Fig. 1). No significant gender differences could be found for any area of competence (data not shown). The ranking of individual items with regard to their respective competence area is shown in Fig. 2. “In need of harmony” (4.36 ± 0.72) reached the highest rank, followed by “Tactfulness” (4.26 ± 0.64), and “Cooperation/Agreeableness” (4.24 ± 0.53). Five of the top ten items belonged to the competence area “Social interactive competences”. Among the bottom ten items, 60 % were found in the competence areas “Mental abilities” and “Sensory abilities”. Particularly low self-ratings were found for “Sanctioning” (2.26 ± 0.93), “Mathematical reasoning” (2.87 ± 1.25), “Risk orientation” (2.90 ± 0.92), “Numeracy” (2.97 ± 1.05), and “Concentration” (3.01 ± 0.89).

**Fig. 3:**
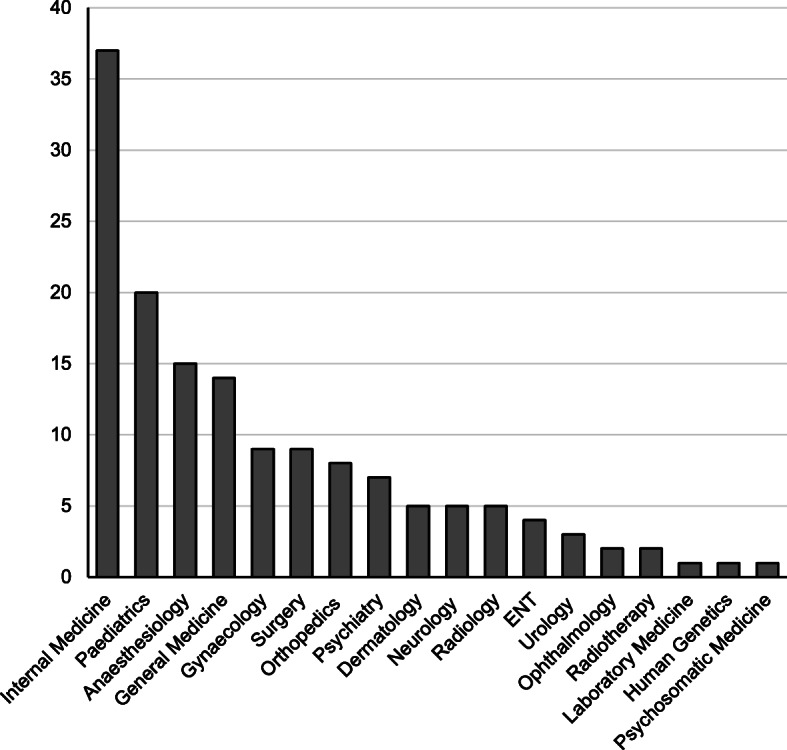
Participants’ first choice of a medical discipline for postgraduate education

**Fig. 4:**
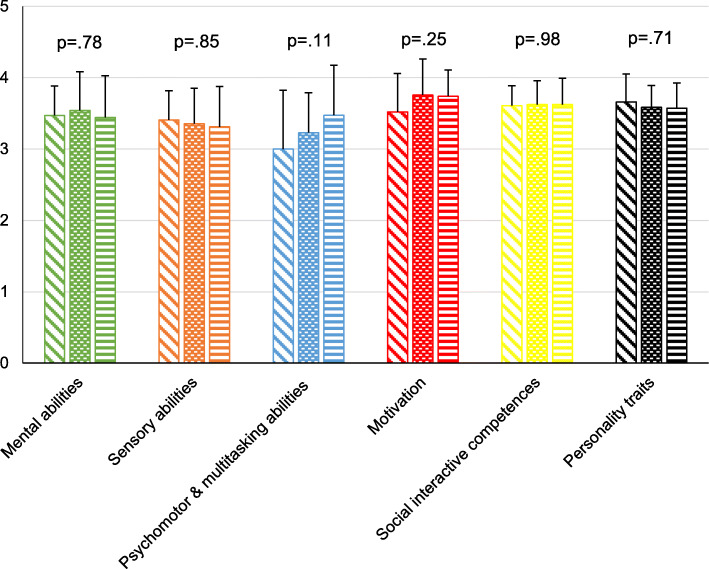
Comparison of competence areas for the postgraduate training choices of anaesthesiology, internal medicine, and paediatrics

A total of 18 different medical specialties for postgraduate training were mentioned by the undergraduate students (Fig. 3). The three most frequently named medical specialties for postgraduate education were internal medicine (25 %), paediatrics (13.5 %), and anaesthesiology (10.1 %). No significant differences (p values ranging from 0.11 to 0.98) were found within the six areas of competence comparing the self-assessments of the medical students who want to choose any of these three specialties for postgraduate education (Fig. 4). While students with a future specialty choice of internal medicine and paediatrics rated “Motivation” on rank 1, “Personality traits” reached the highest rank among students who wish to choose anaesthesiology. “Social interactive competences” reached rank 2 in all groups. Students who wish to choose paediatrics rated “Sensory abilities” lowest, whereas students who want to choose anaesthesiology or internal medicine rated “Psychomotor & multitasking abilities” lowest. Comparing medical students’ competence profiles with the competence profiles defined by physicians working in these three specialties [20], the competence profiles showed 100 % congruence for internal medicine, 33.3 % for paediatrics, and 0 % for anaesthesiology (Table 1). The differences between the highest, and lowest mean of the six competence areas within each group were 0.53 (students) versus 0.98 (physicians) for internal medicine, 0.43 (students) versus 0.93 (physicians) for paediatrics, and 0.66 (students), and 0.75 (physicians) for anaesthesiology.

**Table 1:**
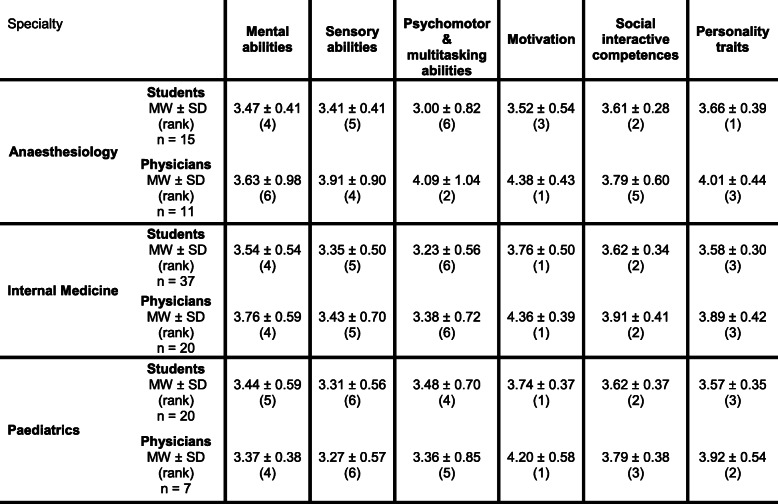
Means of the six competence areas for students and physicians per specialty

## Discussion

To make the right choice for their residency training, medical graduates should know their own abilities, interests and strengths, and compare these with the requirements of the different medical specialties [37, 38]. We found that final-year students rated themselves highest in the competence area “Motivation”. A high level of motivation was found to be necessary to successfully complete undergraduate medical training and become a physician [39, 40]. Furthermore, the competence area “Motivation” also achieved the highest rank by physicians from different medical specialties – irrespective of the specific specialty – as the most important requirement for their daily work [20]. Being motivated for medical learning itself and being interested in a specific specialty seem to be very crucial aspects for the choice of any specialty for postgraduate training. The higher the motivation of medical students, the better the learning quality, learning strategies, perseverance, and the performance in undergraduate medical education [41]. These aspects are important for postgraduate training, too.

The students’ self-assessments also depict, which competences they feel to have acquired during undergraduate education and which competences they still seem to be lacking. The competence facets with the highest ratings mostly belonged to the competence areas “Social interactive competences” and “Personality traits”. The highly rated competence facets “In need of harmony”, “Tactfulness”, “Openness”, and “Agreeableness” reflect aspects that physicians needed for successful communication and good teamwork [42, 43]. During undergraduate medical education, communication and teamwork represent important learning goals, which are prominently integrated in the curriculum [44, 45]. Especially longitudinal and repeated exercises might make students feel more comfortable with facets of competence required for good communication and successful teamwork. The competence facets with the lowest scores belonged to the areas of “Sensory abilities”, “Social interactive competences”, and “Psychomotor & multitasking abilities”. The competence facets “Hearing sensitivity” and “Auditory discrimination”, for instance, are not specifically trained during undergraduate medical education but highly required and exercised in postgraduate anaesthesiology education [46]. “Sanctioning” represents a management and leadership competence [47], which is usually not acquired during undergraduate medical education. The competence facet “Multitasking capacity” received low self-assessment scores, but final-year medical students were also externally rated lowly in this competence facet during a training simulating the first day of residency [48]. An improvement for the transition from undergraduate to postgraduate training with respect to competence has been demanded [49]. In this respect, the modified R-Track could be an additional tool to support curricular planners for undergraduate and postgraduate medical education.

Internal medicine, paediatrics, and anaesthesia have been found in another study to be among the top four specialty choices for postgraduate training in Germany [50]. For these three specialties, we discovered complete to none accordance of the ranks of the six competence areas in the self-assessment of the students, who want to choose the respective specialty for their postgraduate training, with the relevance of the six competence areas for their daily work as assessed by physicians form the respective specialties [20]. However, the difference of the means for the six competence areas was much smaller in the students’ self-assessment than in the physicians’ rating. This suggests that students seem to acquire basic competences in all competence areas [3]. More specific facets of competence from certain competence areas necessary for specific specialties could either be gained during electives in undergraduate training [51, 52] or are very specific for a certain specialty and have to be acquired during postgraduate training [53, 54].

The career decision of physicians for a specialty is of great importance for the long-term structure and safeguarding of the composition of the health workforce [21] and at the same time has a major influence on the job satisfaction of physicians [13]. It could be helpful for undergraduate medical students to be aware of the competence profiles of the different specialties [20] to match their individual self-assessed competence profile with the profile of their desired specialty for postgraduate training, e.g., to identify areas for personal improvement. This could also be useful for undergraduate medical students’ planning of electives and eventually lead to further development of the undergraduate medical curriculum with respect to acquiring specialty specific competences. Additional longitudinal self-assessment might be helpful to identify improvement in certain competences during undergraduate medical education. As differences in self-assessment and rater-based assessments of skills have been observed [55, 56] a combination of students’ self-assessment with the R-Track and assessment of students’ competences by supervisors could be useful to support students’ specialty-specific competence development.

Our study has several limitations. First, a self-assessment is not equivalent to an external assessment. Therefore, an additional external rating of the students’ facets of competence would have provided information about their actual performance in the six areas of competence. Furthermore, only 148 final-year medical students participated. They studied at the same medical school and their participation was voluntary. This limits the generalisability of our study. Another limitation is that the modified R-Track questionnaire – in contrast to its original version [20] – has acceptable Cronbach’s alphas only for three competence areas. Nevertheless, this study provides a first insight about students’ self-assessment in these six competence areas. Comparing the results with physicians’ R-Track results with respect to competence profiles provided first hints for curricular planners to pay attention to certain facets of competence in general. It also helps students to plan their electives concerning their specialty of choice for postgraduate training. Additionally, the comparison of students’ self-assessment versus the R-Track specialty profiles for anaesthesiology, internal medicine, and paediatrics gives first hints for specific facets of competence, which should be acquired or deepened in postgraduate education. Additional studies with larger cohorts of participants from different medical schools are needed to underscore and differentiate our findings. Nevertheless, our data provide evidence that the modified R-Track could provide an additional tool to help undergraduate medical students to make a good choice of the specialty for their postgraduate training.

## Conclusions

With the modified R-Track questionnaire, we were able to define self-assessed competence profiles of final-year undergraduate medical students. The six competence areas showed very similar means with the competence area “Motivation” reaching the highest rank. Regarding the individual facets of competence, the top ten and bottom ten seem to depict these facets of competence as being represented or not represented in the undergraduate medical curriculum, respectively. Comparing the students’ R-Track self-assessment and their choice of specialty for postgraduate training with physicians’ R-Track assessment from the respective specialties, discrepancies in expression and requirement of the competence areas became evident. Students’ use of the modified R-Track questionnaire could support them in planning their undergraduate education with respect to the facets of competence, which are particularly required in their specialty of choice for postgraduate training. Combined with medical students’ external assessment, results from the modified R-Track questionnaire could provide curricular planners with useful information how to design learning opportunities for general and more specialty-specific facets of competence.

## Supplementary Information

Additional file 1: **Supplement 1. **Competence facets of the self-assessment R-Track Questionnaire.

## Data Availability

All data and materials are available from the manuscript and from the corresponding author upon request.

## Abbreviations

ANOVA: Analysis of variance
F-JAS: Fleishman job analysis survey
ID code: Identification code
LSD: Least significant difference test
NRMP: National resident matching program
ROLs: Rank order lists
R-Track: Requirement-Tracking
SPSS: Statistical package for the social sciences
