# Supplementary material for: Final-year medical students’ competence profiles according to the modified requirement tracking questionnaire
Source: BMC Med Educ. 2021 Jun 5;21:319. doi: 10.1186/s12909-021-02728-2 (PMC8178874; doi:10.1186/s12909-021-02728-2)
Supplement: Supplementary file 1 — Supplement 1. Competence facets of the self-assessment R-Track Questionnaire. [file 12909_2021_2728_MOESM1_ESM.docx]

**Supplement 1: Competence facets of the self-assessment R-Track Questionnaire**

| **No.** | **Competence facets** | **No.** | **Competence facets** |
| --- | --- | --- | --- |
| 1 | Expertise | 33 | Written expression |
| 2 | Risk orientation | 34 | Sense of humour |
| 3 | Diplomacy | 35 | Manners and common decency |
| 4 | Emotional stability | 36 | Clarity of speech |
| 5 | Persuasiveness | 37 | Norms and values orientation |
| 6 | Tactfulness | 38 | Coaching and Mentoring |
| 7 | Creativity | 39 | Orientation towards clients and/or patients |
| 8 | Openness to novelty | 40 | Sovereignty |
| 9 | Flexibility | 41 | Endurance |
| 10 | Independence and autonomy | 42 | Logical reasoning |
| 11 | Prudence | 43 | Numeracy |
| 12 | Thoroughness | 44 | Memory capacity |
| 13 | Reliability and discipline | 45 | Perceptual speed |
| 14 | Stress resistance | 46 | Comprehension |
| 15 | Resistance to monotony | 47 | Perceptual range |
| 16 | Openness to other people/cultures | 48 | Near vision |
| 17 | Tolerance to frustration | 49 | Range of field vision |
| 18 | Achievement motivation | 50 | Auditory discrimination |
| 19 | Coordination and decision making | 51 | Visual imagination |
| 20 | Verbal expression | 52 | Hearing sensitivity |
| 21 | Presentation | 53 | Facility for languages |
| 22 | Conflict management | 54 | Selective attention |
| 23 | Sanctioning | 55 | Reading comprehension |
| 24 | Cooperation / Agreeableness | 56 | Problem comprehension |
| 25 | Honesty | 57 | Verbal understanding |
| 26 | Modesty | 58 | Psychomotor coordination |
| 27 | Willingness to help | 59 | Multitasking capacity |
| 28 | In need of harmony | 60 | Mathematical reasoning |
| 29 | Delegation / Delegating | 61 | Concentration |
| 30 | Structuring Information | 62 | Spatial orientation |
| 31 | Self-confidence | 63 | Spatial visualization |
| 32 | Sociability |  |  |
